# Critical review of indicators, metrics, methods, and tools for monitoring and evaluation of biofortification programs at scale

**DOI:** 10.3389/fnut.2022.963748

**Published:** 2022-10-13

**Authors:** Santiago Rodas-Moya, Francesca M. Giudici, Bho Mudyahoto, Ekin Birol, Stephen R. Kodish, Carl Lachat, Taymara C. Abreu, Alida Melse-Boonstra, Karin H. van het Hof, Inge D. Brouwer, Saskia Osendarp, Edith J. M. Feskens

**Affiliations:** ^1^Division of Human Nutrition and Health, Wageningen University, Wageningen, Netherlands; ^2^HarvestPlus, c/o International Food Policy Research Institute, Washington, DC, United States; ^3^Edmund A. Walsh School of Foreign Service, Global Human Development Program, Washington, DC, United States; ^4^Department of Nutritional Sciences and Biobehavioral Health, Pennsylvania State University, University Park, PA, United States; ^5^Department of Food Technology, Safety and Health, Faculty of Bioscience Engineering, Ghent University, Ghent, Belgium; ^6^Department of Epidemiology and Data Science, Amsterdam UMC, Location VUmc, Amsterdam Public Health Research Institute, Amsterdam, Netherlands; ^7^The Micronutrient Forum, Washington, DC, United States

**Keywords:** biofortification programs, monitoring and evaluation frameworks, indicators, metrics, methods, tools

## Abstract

Sound monitoring and evaluation (M&E) systems are needed to inform effective biofortification program management and implementation. Despite the existence of M&E frameworks for biofortification programs, the use of indicators, metrics, methods, and tools (IMMT) are currently not harmonized, rendering the tracking of biofortification programs difficult. We aimed to compile IMMT for M&E of existing biofortification programs and recommend a sub-set of high-level indicators (HLI) for a harmonized global M&E framework. We conducted (1) a mapping review to compile IMMT for M&E biofortification programs; (2) semi-structured interviews (SSIs) with biofortification programming experts (and other relevant stakeholders) to contextualize findings from step 1; and (3) compiled a generic biofortification program Theory of Change (ToC) to use it as an analytical framework for selecting the HLI. This study revealed diversity in seed systems and crop value chains across countries and crops, resulting in differences in M&E frameworks. Yet, sufficient commonalities between implementation pathways emerged. A set of 17 HLI for tracking critical results along the biofortification implementation pathway represented in the ToC is recommended for a harmonized global M&E framework. Further research is needed to test, revise, and develop mechanisms to harmonize the M&E framework across programs, institutions, and countries.

## Introduction

Micronutrient malnutrition affects ∼2 billion people worldwide (1, 2); it contributes to poor child growth, intellectual impairment, increased risk of morbidity and mortality, and is highly prevalent in food-insecure settings in low-and middle-income countries (LMIC) (3). Biofortification, an agriculture-based method of increasing the density of micronutrients of staple crops through selective plant breeding and agronomic techniques, can play a crucial role in addressing micronutrient malnutrition (4, 5) since it reaches populations in remote rural areas and could potentially reach urban consumers as well (6, 7).

Under the leadership of HarvestPlus, crop-breeding programs at CGIAR centers, national agricultural research systems (NARS), private, public, and community-based seed producers, and farmers have co-developed, tested, and released more than 400 biofortified varieties of 11 different crops worldwide (8). The efficacy of biofortified food to improve nutritional and health outcomes has been demonstrated consistently in the past 15 years, especially for vitamin A enriched crops, iron beans, and iron-pearl millet (4, 6), and cost-effectiveness has been demonstrated for vitamin A enriched crops (9).

Given its promise for improving diets, biofortification is at a tipping point to go to scale to replace currently grown staples with low nutrient density (10), contributing to food system transformation without changing consumer eating behaviors (7). For this, national governments, non-governmental organizations (NGOs), the private sector, the UN, and international financial institutions will need to invest more in context-specific biofortification programs (10, 11) with an increased diversity of stakeholders across geographies. As biofortification scaling efforts gain momentum (10), so does the need for a monitoring and evaluation (M&E) framework with a set of harmonized indicators metrics, methods, and tools (IMMT) that allow the measurements of key results of biofortification programs across countries, regions, and organizations.

Sound M&E systems are needed to generate quality data on program performance to inform learning and adaptive program management for effective implementation and evidence-based policymaking (12). Despite the existence of comprehensive M&E systems for many of the current biofortification programs, their focus has primarily been on project-level management, and the IMMTs are currently not harmonized across countries, crops, and implementors. Furthermore, the existing M&E frameworks for biofortification interventions are primarily reported in gray literature, such as donor reports and institutional publications. Hence, there is a need to review and document the tried-and-tested, found-to-work, and common elements of the existing M&E frameworks to facilitate harmonization across programs, crops, countries, and organizations.

**FIGURE 1:**
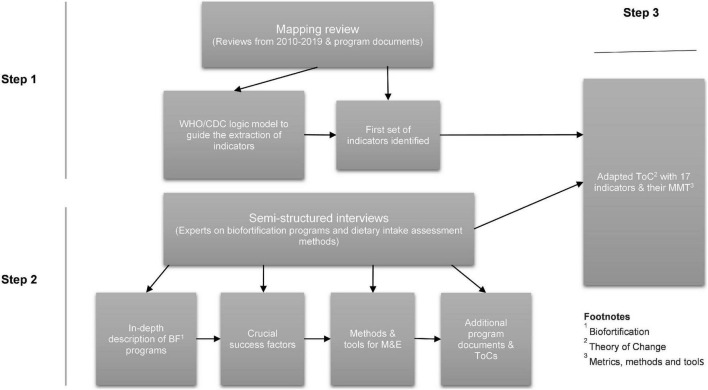
Overview of the multi-phase, iterative process research approach.

In a multi-phase, iterative process, we aimed to review M&E frameworks of biofortification programs implemented to date, and to recommend a subset of high-level indicators (HLI) for a harmonized global M&E framework to track program progress along a generic implementation pathway. We conducted (1) a mapping review to compile IMMT for biofortification programs; (2) semi-structured interviews (SSIs) with biofortification programming experts (and other relevant stakeholders) to contextualize findings from step 1; and (3) compiled a generic biofortification program Theory of Change (ToC) to use it as an analytical framework for selecting the HLI (Figure 1). This study responds to the collective call to strengthen monitoring and evaluation efforts to support healthy and sustainable food systems—“The Accountability Pact” (13)—and to the need to use rigorous monitoring to guide food system transformation in the countdown to the 2030 global goals (12). We expect to fill an important gap in the literature concerning best-bet M&E frameworks for biofortification programming.

## Step 1—Mapping review

We focused on published reviews and official program documents that contained detailed descriptions of biofortification programs, M&E frameworks, and IMMT. MEDLINE, Cochrane Library, Web of Science, and Scopus were searched to retrieve reviews that met the inclusion criteria (Table 1). Six building blocks of strings were developed for MEDLINE by adapting the search strategy of Garcia-Casal et al. (14). The search syntax included MeSH terms in the title and abstract fields (Supplementary Data). The building blocks and strings created for MEDLINE were adapted for searches in the other databases. The final search results were imported into EndNote; duplicates were removed automatically. We also accessed project reports from the Inclusive and Sustainable Value Chains and Food Fortification projects (EuropeAid/151093/DH/ACT/Multi) shared by the European Commission’s Food Fortification Advisory Service. Based on the inclusion and exclusion criteria (Table 1), two investigators (TCA and SRM) independently selected reviews and program documents for further use. Any disagreements between the investigators were resolved through consultation with another researcher from the team (CL).

**TABLE 1:** Inclusion and exclusion criteria for the mapping review.

Inclusion criteria	Exclusion criteria
● Reviews on biofortification programs implemented in LMIC^a^ in the last ten years ● Reviews on biofortification programs that include staple crops biofortified with one or more nutrients using conventional breeding techniques or agronomic practices (e.g., cassava, sweet potato, beans, pearl millet, rice) ● Biofortification program documents, such as reports, logic models, M&E manuals, technical monographs, M&E tools or leaflets, and videos. ● Reviews, other studies, and gray literature written in English, Spanish, or French	● Literature on biofortification programs implemented in HIC^a^ ● Literature on biofortification using genetically modified organisms ● Published literature and gray literature written in languages other than English, Spanish, or French. ● Full-text unavailable ● Reviews published more than ten years ago (since 2010).

^*a*^Low- and Middle-Income Countries and High-Income Countries as defined by the World Bank (57).

To guide the identification of indicators for M&E from the published and gray literature, we adapted the WHO/CDC logic model for implementing micronutrient interventions in public health (15) to represent the underlying implementation processes of biofortification programs (Supplementary Figure 1) and identified relevant input, output, outcome, and impact indicators. Next, two authors (TCA and SRM) developed two data charting forms in Microsoft Excel 2016 to extract the IMMT from the reviews and gray literature. CL pilot-tested the charting forms. No changes were made after the pilot test, and input, activity, output, outcome, and impact indicators were extracted accordingly.

## Step 2—Semi-structured interviews with biofortification programming experts and other relevant stakeholders

The SSIs with biofortification programming experts aimed to contextualize the findings from the mapping review by obtaining a generic description of biofortification programs, key factors limiting/enabling their success, M&E frameworks used, and IMMT, as well as methods for assessing coverage and consumption of biofortified foods. SSI guides were developed accordingly with guiding semi-structured questions and detailed probes to gain in-depth information on key topics of inquiry (16).

Nine biofortification programming experts and one food systems expert were recruited through snowball sampling (17) and were invited for an (online) interview. Two experts, EB and BM, are also coauthors of this paper. The participants’ expertise in biofortification programming covered Africa, Asia, Latin America, and the Caribbean regions, and their collective programming expertise included global to regional, and local levels of biofortification programming. In addition to the biofortification (*n* = 9) and food systems (*n* = 1) experts, two experts with extensive food intake assessment experience were interviewed to obtain further details on the challenges and opportunities of conducting dietary intake assessments in LMIC. The latter interviews enabled an understanding of the most suitable methods for assessing consumption of biofortified foods and potential adaptations that could be made to existing diet quality scores. Table 2 shows participants’ affiliations.

**TABLE 2:** Characteristics of SSI participants.

Organization	Generic role	No. of participants
HarvestPlus	Agriculture and nutrition strategy	2
	Global M&E and impact assessment	2
	Regional M&E	1
CIAT	Agricultural research	1
CIP	Agriculture and nutrition strategy	1
	Local and regional implementation and M&E expertise	2
Johns Hopkins	Global food policy & ethics	1
Harvard T.H. Chan School of Public Health	Expertise in dietary intake assessment methods in LMIC	1
Intake	Expertise in dietary intake assessment methods in LMIC	1
Total		12

On average, each interview lasted 75 min and was conducted in English. The interviews were digitally recorded and transcribed *verbatim* by two research assistants for subsequent thematic analysis. The content of the transcripts was spot-checked by FMG and SRM with the original audios to ensure content fidelity. Data were collected over five weeks from March to June, 2021 until data saturation was reached among key themes (16, 18).

During the conduct of the SSIs, some participants from HarvestPlus shared additional program documents: an unpublished generic biofortification ToC from 2016 (19), three unpublished country- and crop-specific ToCs (20–22), and the published ToC for the Commercialization of Biofortified Crops Program led by HarvestPlus and the Global Alliance for Improved Nutrition (GAIN) (23). The latter was adapted from an unpublished harmonized ToC developed by a collective working on developing a harmonized monitoring, evaluation, learning, and impact assessment system (MELIAS) for large-scale biofortification programs from HarvestPlus, CIP, and GAIN. SSI participants from HarvestPlus also shared a set of 19 M&E indicators anchored to the MELIAS biofortification ToC. This was part of an internal HarvestPlus M&E manual (24). Finally, a CIP participant shared an M&E tool for orange-fleshed sweet potato (OFSP) interventions in sub-Saharan Africa (25), from which one indicator was extracted. These and other documents reviewed for further analysis are presented in Table 3.

**TABLE 3:** List of additional documents received from SSI participants from HarvestPlus and CIP.

Name of document	Type of document	Organization	Food vehicle	Micronutrient
Strategic Brief: Catalyzing the Scale-up of Crop Biofortification (42)	Strategic brief	HarvestPlus	All crops	Various
Biofortification Indicator Definition Tables (24)	Manual	HarvestPlus	All crops	Various
HarvestPlus varietal development and delivery models for biofortified crop varieties (43)	Working paper	HarvestPlus	All crops	Various
Harmonized Theory of Change for Scaling up Biofortification developed by HarvestPlus, CIP, and GAIN (23)	Impact pathway	HarvestPlus, CIP, GAIN	All crops	Various
Generic biofortified crop ToC (19)	Impact pathway	HarvestPlus	All crops	Various
Vitamin A Cassava ToC, generic (20)	Impact pathway	HarvestPlus	VAC	Vitamin A
Vitamin A Cassava, ToC, Nigeria (21)	Impact pathway	HarvestPlus	VAC	Vitamin A
Vitamin A Maize ToC, Zambia (22)	Impact pathway	HarvestPlus	Maize	Vitamin A
HarvestPlus Technical Monograph Series: 4—Analyzing the Health Benefits of Biofortified Staple Crops by Means of the Disability-Adjusted Life Years Approach: a Handbook Focusing on Iron, Zinc, and Vitamin A (53)	Technical monograph	HarvestPlus	All crops	Various
Tools and Techniques for Monitoring and Evaluating Sweet potato Interventions in Sub-Saharan Africa: A Practitioner’s Toolkit (25)	Manual	CIP	OFSP	Vitamin A

### Data analysis of semi-structured interviews and program documents

Thematic analysis followed procedures suggested by Huberman et al. (18). First, SRM and FMG read the program documents and interview transcripts several times to gain a holistic view of the data set. Second, a codebook with 25 categories of information that served as a framework for analysis was developed and cross-checked by another researcher from the team (SRK). The initial codes were applied to the text in program documents and SSI transcripts to tag content based on meaning (first coding cycle). FMG coded all program documents, and the interview transcripts were coded in duplicate by SRM and FMG. Third, through a second coding cycle, the 25 codes were clustered and merged into eight pattern codes (i.e., thematic areas). Interrelations between pattern codes were then examined, and three primary themes aligned with the study aim were extracted for theory building. Exemplar quotations that best described the topic of interest were selected to illustrate the findings. Dedoose (26) software version 8.3.47 was used for data management and analysis of secondary sources of information and interview transcripts.

## Step 3—Compiling a generic Theory of Change and selecting indicators, metrics, methods, and tools for biofortification programs

The implementation processes of biofortification programs described by the SSI participants were compiled into a narrative structure and subsequently represented in an initial draft ToC. This draft was then compared to the ToCs from Table 3, adapted to add detail and ensure that common elements of biofortification interventions were represented in the compiled ToC. Next, the indicators selected from the mapping review (36 indicators) were merged with the new set of 19 indicators (24) anchored to the ToC developed by the MELIAS group (27), one indicator extracted from the CIP M&E manual (25), and one indicator recommended by three HarvestPlus participants in SSIs into a final list of 57 indicators (Table 4). Indicators that, from our perspective, had similar or overlapping definitions with those of the list of 19 indicators (24) were eliminated (Table 4, 13 indicators marked in red). Furthermore, as we aimed to keep only high-level indicators that reflected critical implementation stages of biofortification programs across the ToC, input and activity level indicators were excluded (four input and six activity indicators marked in orange). A total of 34 indicators (marked in black, blue, and green) were kept to select a final subset of HLI. Using the ToC of this study as an analysis framework, the authors selected the final list of HLI.

**TABLE 4:** Total number of indicators (*N* = 57), extracted from the mapping review (*n* = 36 orange, black and red indicators) merged with the 19 indicators (in green) anchored to the generic ToC from the MELIAS group, one indicator recommended by three HarvestPlus experts, and one indicator extracted from the CIP monitoring and evaluation manual (in blue).

1. Input	2. Activities	3. Outputs	4. Outcomes	5. Impact
1.1. Financial resources (33)	**Development of tools for data collection**	**Crop development**	**Production of biofortified crops**	**Nutritional impact**
	2.1. Number of tools developed (33)	3.1. Number of varieties (crop development lines) under on-station research (33)	4.1. Proportion/number of farm households that acquired^b^ planting material (24)	5.1. Proportion of individuals in target population group whose micronutrient intake status shifts from nadequate to adequate due to the consumption of biofortified food(s) over time (24)
1.2. Human Resources (33)	**Information sharing about biofortified crops in platforms and events**	3.2. Number of varieties field-tested (33)	4.2. Proportion of households reached with planting material through farmer to farmer sharing (33)	i5.2. Proportion of the estimated average requirement delivered disaggregated by crop and target group (33)
	2.2. Number of information sharing events for disseminating information about biofortified crops and food products (33)			
1.3. Parental breeding lines (33)	2.3. Number of people attending information sharing events about biofortified crops and food products (33)	3.3. Proportion of crop varieties released that are biofortified (24)	4.3. Number of households reached through the acquisition of planting material from the seed market (33)	5.3. Change in the prevalence of inadequate intake of target micronutrients in project intervention areas (33)
1.4. Intellectual property (33)	**Development of materials for education information and communication (IEC)**	3.4. Proportion of crop varieties with minimum micronutrient level released^a^	4.4. Percent of farmers who planted biofortified crops received from the program (4, 30, 33)	5.4. Number of disability-adjusted life years averted (33)
	2.4. Number of information, education, and communication (IEC) materials developed (33)			
	**Development of platforms for multiplication and delivery of planting material**	3.5. Number of varieties released (33)	4.5. Area planted with biofortified crops by farmers (33)	5.5. Change in prevalence of dietary diversity or diet quality Score between baseline and end of project (25)
	2.5. Number of commercial and public platforms able to multiply and deliver planting material (31)			
	2.6. Number of community-based producers that multiply and deliver planting material (31)	3.6. Proportion of improved planting material (seed) that is biofortified (24)	4.6. Proportion of crop area that is allocated to biofortified varieties (24)	
		3.7. Quantity of planting material produced (per type of producer) that is available in warehouses or the fields for the next planting season (33)	4.7. Percent share of area planted with biofortified crops (33)	
		**Delivery of planting material and production of biofortified crops**	4.8. Proportion of crop harvest that is of biofortified varieties (24)	
		3.8. Quantity of planting material acquired by farm households (24)		
		3.9. Number of households reached through the delivery of certified planting material for production purposes (disaggregated by gender) (4, 29, 33)	**Distribution of biofortified foods through different channels**	
			4.9. Proportion of farm households that sell biofortified crops/food (24)	
		3.10. Quantity of planting material delivered to farmers (33)	4.10. Proportion of farmers selling biofortified foods (32)	
		3.11. Proportion/number of households that are growing biofortified crop varieties (24)	4.11. Proportion of raw food in the market that is biofortified (24)	
		3.12. Quantity of biofortified crops harvested/produced (in MT) (4, 28, 30)	4.12. Proportion of raw input for processed foods that is biofortified (24)	
		3.13. Proportion of seed in institutional seed/input distribution programs that is biofortified (24)	4.13. Proportion of prepared or processed food products available in the market that contain a biofortified food (24)	
		**Capacity development**	4.14. Proportion of staple foods in institutional food distribution programs that is biofortified (24)	
		3.14. Number of people trained in biofortification related topics (24)		
		3.15. Number of people trained (33)	4.15. Percent of food market share of biofortified foods (33)	
			**Awareness, availability, and consumption of the biofortified foods in farm and non-farm households** 4.16. Consumption of the food (34)	
			4.17. Proportion of people that are aware of biofortified crops/foods (24)	
			4.18. Awareness of the biofortified food (34)	
			4.19. Availability of the biofortified food (34)	
			4.20. Consumption of the biofortified food (ever) (34)	
			4.21. Consumption of the biofortified food (current) (34)	
			4.22. Proportion/Number of individuals in target population group who consumed (in any amount) the biofortified food (24)	
			4.23. Proportion of target group who consume biofortified food products (32, 33)	
			4.24. Amount of the biofortified food consumed daily among target population group (24)	
			4.25. Average intake of biofortified food among target groups (28, 29)	
			4.26. Frequency of consumption of biofortified food during the past seven days among the target groups (32)	
			4.27. Number of policy/plan documents that mention biofortification as a strategy for improving (24)	

**Total number of indicators for subsequent selection of high-level indicators after removing orange and red indicators**				

0	0	10	19	5

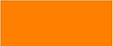	Indicators of input and activity level were not considered for the final selection of high-level indicators. We intended to keep high-level indicators focusing on the measurement of results of critical implementation stages of biofortification programs along the compiled ToC.			
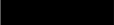	Indicators kept for the final selection of high-level indicators.			
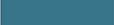	Indicator recommended by three HarvestPlus experts and indicator obtained from CIP M&E manual, respectively.			
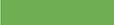	Indicators belonging to the list of 19 indicators developed by HarvesPlus, CIP, and GAIN (24) kept for subsequent selection of high-level indicators.			
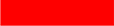	Indicators that were not considered for selecting the high-level indicators because of similarity or overlap with other indicators.			

^*a*^This Indicator focuses on the number of varieties that have been biofortified to a minimum recommended level required to have the expected impact for correcting micronutrient deficiencies. It was recommended by three experts on BF programming from HarvestPlus.

^b^“Acquired” refers to planting material distributed, given, or sold to farmers. There are three ways of acquiring seed depending on the source and means of payment: (1) Farmers can pay cash or barter trade to get seed from the seed market; (2) Farmers can receive free seed as promotional packs; and (3) Farmers can get recycled seed from fellow farmers through farmer to farmer sharing, or obliged sharing, e.g., payback. For this case, the indicators 4.2–4.4 were removed because they were contained in indicator 4.1.

## Ethical considerations

Participation in the study was voluntary. Before the interviews, potential participants received a detailed explanation (verbal and written) about the purpose of the study and were informed that the data would be used for research purposes. SSI participants gave verbal consent to record the interviews. As data collection methods were primarily desk-based and contact with participants was minimal (online interviews), approval from an institutional review board was not sought.

## Mapping review (Step 1)

The mapping review resulted in three published reviews fulfilling the eligibility criteria (4, 28, 29) and four program documents (30–33). An additional scientific paper (34) recommended by experts was also included (Table 5). Although not a review, this study aimed to develop and test methods and indicators for assessing awareness and household coverage of biofortified foods in Rwanda. It yielded five outcome indicators with their respective data collection methods. From the three reviews (4, 28, 29), we identified four outcome indicators and two output indicators.

**TABLE 5:** List of documents included in the mapping review.

Document	Type of document	Publication Journal/Organization	Food vehicle	Micronutrient
Bouis HE, Saltzman A. *“Improving nutrition through biofortification: A review of evidence from HarvestPlus, 2003 through 2016”* (4)	Review	Global Food Security Journal	Various crops	Various
Laurie S, Faber M, Adebola P, Belete A. *“Biofortification of sweet potato for food and nutrition security in South Africa”* (28)	Review	Food Research International	Sweet potato	Vitamin A
Lockyer S, White A, Buttriss JL. *“Biofortified crops for tackling micronutrient deficiencies – what impact are these having in developing countries and could they be of relevance within Europe?”* (29)	Review	Nutrition Bulletin	Various crops	Various
Petry N, Wirth JP, Friesen VM, Rohner F, Nkundineza A, Chanzu E, et al. *“Assessing the Coverage of Biofortified Foods: Development and Testing of Methods and Indicators in Musanze, Rwanda.”* (34)	Article	Current Developments in Nutrition	High-iron beans; OFSP	Iron; Vitamin A
Baluu Tim-Maring-Ngo Project - Reducing micronutrient deficiencies of women and children in The Gambia through sustainable and integrated approaches to food fortification. In Final Narrative Report. (30)	Report	European Union; FAO; United Purpose	OFSP	Vitamin A
Improving Food Security and Nutrition in the Gambia through Food Fortification. Interim Project Report (FOOD/2016/380-042). (31)	Report	European Union; FAO; United Purpose	OFSP	Vitamin A
Sustained Diet Quality Improvement by Fortification with Climate-smart, Nutrition-Smart Orange-fleshed Sweetpotato in Southern Nations, Nationalities and Peoples’ Region (SNNPR), Ethiopia; also known as Quality Diets for Better Health (QDBH). In Annual Project Report (FOOD/2016/380-038). (32)	Report	CIP; People in Need; Emory University; European Union	OFSP	Vitamin A
Indicator Reference Manual (IRM) (33)	Manual	HarvestPlus	Various crops	Various

From the gray literature, three reports that focused on tracking the progress of biofortification programs in tackling micronutrient deficiencies in the Gambia (30, 31) and Ethiopia (32) were reviewed, from which we extracted four outcome, one output, and two activity indicators. In addition, from the HarvestPlus Indicator Reference Manual (33), we extracted four input, four activity, seven output, seven outcome, and three impact indicators. Indicators with similar definitions were grouped into a single indicator citing the sources. When available, the indicators were extracted with their metrics, methods, and tools for constructing their numeric values. Overall, the mapping review yielded 36 unique indicators presented in Table 4 (indicators marked in orange, black, and red) with their respective sources.

## Semi-structured interviews with biofortification programming experts and other stakeholders (Step 2)

The findings of the SSIs were grouped into three main themes, as described below.

### Theme 1—Generic Description of biofortification programs

Most participants described the biofortification programs in four stages. Those descriptions were also comparable to the biofortification interventions described in the reviews (4, 28, 29) and program documents listed in Tables 3, 5. A generic description of biofortification programs centered around common elements of successful programs is presented below in four stages.

*Stage 1: Breeding and releasing biofortified crop varieties*—All participants indicated that the initial development of biofortified varieties of crops was primarily carried out at the CGIAR centers at global and regional levels. In LMIC, the CGIAR shares biofortified parental lines with NARS for inclusion in national breeding programs for further adaptive breeding and field testing. Subsequently, the NARS, or other national varietal release authority, may approve the release of tested biofortified varieties with competitive agronomic traits if they meet predetermined threshold micronutrient levels and other agronomic characteristics as required by the national authorities. The released varieties may then be licensed to public and private companies (small and medium scale) for multiplication and subsequent distribution to farmers. Most participants described biofortification breeding as a “*dynamic*,” “*never-ending process,” “constantly searching for high-yielding, pest-resistant, and climate-resilient varieties where breeding for higher micronutrient levels—enough to have a significant impact on micronutrient deficiencies—is mainstreamed into the existing breeding programs.”* Most participants considered investing in capacity strengthening at the NARS level for breeding and testing new biofortified varieties crucial for successfully sustaining biofortification in public and private breeding programs over time.

*Stage 2: Multiplying, introducing, and distributing planting material of biofortified crop varieties to farmers*—Most participants explained that multiplication and distribution of planting material are carried out by private, public, or community-based planting material producers, depending on the type of crop. Grain crop seeds for hybrid and open-pollinated varieties (OPV), such as maize and pearl millet, are usually licensed to commercial private and/or public seed companies for subsequent multiplication and marketing. In contrast, vegetatively propagated crops (VPC), such as sweet potato and cassava, are usually multiplied and distributed by community-based stem/vine multipliers.

Depending on the competitiveness of the planting material, there is a mixed seed production and distribution system for OPVs and self-pollinating biofortified varieties of crops (SPV), such as beans, rice, and wheat. Private seed companies prefer to produce and market highly competitive varieties (e.g., hybrid and OPVs), while public sector and NGO support are needed for less competitive varieties with high levels of micronutrients. All participants explained that the crop type and seed systems also define the seed commercialization pattern. Depending on the crop, private seed companies, public multipliers, NGOs, farmer organizations, including women’s groups, or a combination of two or more of these are crucial to scaling the multiplication of biofortified planting material and hence availability thereof to smallholder farmers.

*Stage 3: Scaling production and utilization of biofortified foods*—All participants indicated that scaling will be the focus of most national and global programming in the coming decade. Strengthening all value chain actors, from breeders to farmers, aggregators, processors, retailers, and other service providers, is a crucial cross-cutting aspect of program implementation activities to integrate biofortification into seed and food systems in a sustainable way. Implementing behavior change communication strategies to accelerate awareness and adoption of biofortified crops, fostering good pre and post-harvesting practices, and facilitating access to and promoting utilization of biofortified planting material among farmers are crucial in this stage. Subsidies, partial or complete, and free or low-cost demonstration kits/trial packs can effectively foster the adoption of biofortified crops by farmers. Some biofortification programs also establish linkages between farmers and different market options, such as food processors, to help farmers and aggregators sell their products as ingredients for processed foods.

Most participants also highlighted the role of the public sector, NGOs, and humanitarian organizations in scaling access to biofortified planting material and foods and using biofortified foods for emergency response. For example, CIP participants indicated that they work with the World Food Programme to include OFSP in food security programs in refugee camps in northern Ghana, northern Uganda, and northern Kenya. Other examples include involving the public sector in distributing OFSP in school feeding programs in Nigeria and Ghana. Most participants also indicated that behavior change strategies to promote the utilization of biofortified crops and foods among potential consumers in rural and urban settings are crucial in the scaling phase.

*Stage 4: Integrating biofortification in local food systems—*A participant described the mainstreaming of biofortification into food systems as follows:
*“… biofortification needs to be mainstreamed into national policies, plans, and breeding programs. The new release of competitive biofortified varieties is actively ongoing; the varieties are tested by NARS, released for production by national authorities, and adopted by farmers, and the production of biofortified crops increases continuously. As a result, biofortified foods are mainstreamed into the agricultural and food sectors; consequently, consumption of biofortified food increases, contributing to relieving micronutrient deficiencies and improving the quality of diets.”*

*Interview with senior expert, Washington, DC, March 2021*

Furthermore, some participants indicated that defining standards with ranges of micronutrient content for biofortified planting material and food manufactured with biofortified ingredients is crucial to ensure that biofortified crop varieties and foods meet the expected micronutrient content in the future.

### Theme 2—M&E frameworks for biofortification programs

From the SSIs, two application scenarios emerged for M&E of outcome (adoption) and coverage of biofortification programs and consumption of biofortified foods: nationally representative surveys and sentinel site monitoring surveys. All participants indicated that nationally representative surveys are warranted only when the program has reached sufficient maturity and coverage and recommended the integration of biofortified crops and foods into production and consumption modules of existing nationally representative data collection systems (e.g., National Crop Surveys, National Demographic and Health Surveys) to optimize costs and sustainability of M&E of production, coverage, and consumption of biofortified crops and foods. On the contrary, some participants indicated that sentinel site surveys could be used for monitoring pilot stage or “nascent programs” or in cases of budget constraints for M&E. In a sentinel surveillance system, data are regularly reported from a sample of pre-selected sites (units of programming such as district or local government authority) purposefully selected to represent the population of the areas where the programs are implemented (35). The required sample size for sentinel site surveys is smaller and can prioritize program intervention areas. Therefore, these are less costly and simpler to conduct than nationally representative surveys (35).

### Theme 3—Compiling methods, metrics, and tools for constructing indicators

The following sections discuss the methods and tools to assess the coverage and consumption of biofortified crops described in the SSIs. This section specifically concerned diversity and diet quality scores, as used by CIP and HarvestPlus in biofortification programs (and others in generic nutrition-specific and nutrition-sensitive interventions).

#### Methods for assessing coverage and consumption of biofortified crops/foods

A lack of agreement emerged among participants on the most appropriate methods for assessing coverage and consumption of biofortified foods. Some participants indicated a preference for traditional consumption surveys such as 24-hour recalls (24hR) or Food Frequency Questionnaires (FFQ). In contrast, others suggested alternative methods, such as mathematical modeling of Household Consumption and Expenditure Surveys (HCES) data to estimate apparent food consumption and micronutrient intake (before and after biofortification of the staple[s] consumed) based on the adult male equivalent formula, as proposed by the Food and Agriculture Organization (FAO) (36, 37).

Concerning HCES, some participants voiced concern that these may produce inaccurate estimates of individual food consumption and nutrient intakes, primarily because they do not consider intra-household food sharing practices or foods consumed away from home. In contrast, others argued that considering the complexity of conducting 24hR or FFQ, their estimates are also inaccurate. The most salient examples of sources of inaccuracy mentioned for 24hR were:

• standardized portion sizes are seldom available for specific LMIC contexts;
• extensive training and experience are required to collect, process, and analyze data from 24hR and FFQ. Such training is often lacking, leading to inaccurate data collection processes and poor data quality.
• For FFQ, appropriate food lists tailored to the context and population group are needed, requiring initial formative research and validation against 24hR and is therefore even more rare in LMIC.

Other mentioned barriers to undertaking 24hR and FFQ were the time required for data collection, processing, and analysis of data, cost, and complex logistics and the availability of up-to-date and accurate food composition tables in LMIC.

In light of the above limitations, one participant explained that apparent food consumption estimated from HCES is sufficient to estimate the coverage and consumption of biofortified foods, as long as biofortified foods are included in the surveys as specific categories of food. The participant also indicated that the advantage of HCES is that food acquisition or consumption data are regularly collected at the household level in multiple LMIC and that the data are publicly available in most cases. Another participant indicated that including biofortified foods in the food acquisition and consumption lists of HCES would allow making sufficiently robust estimates of coverage and consumption of biofortified foods for routine program monitoring.

#### Diet diversity and diet quality scores

Given the complexity and cost of undertaking dietary intake surveys, some metrics have been developed to simplify diet quality assessment. One of them is the minimum dietary diversity score for women (MDD-W), a dichotomous indicator that assesses dietary diversity and estimates nutrient adequacy among women of reproductive age (WRA) (38). The MDD-W’s score ranges from 0 to 10 (0–10 food groups consumed in the past 24 h), with a cut-off of <5 indicating the inadequacy of micronutrient intake (39). However, some participants pointed out that the MDD-W was not designed to assess the consumption of specific foods, such as biofortified foods, nor to quantify nutrient intake from food consumption. Hence, the MDD-W would provide limited actionable information for monitoring and adjusting biofortification programs:

*“…Diet indicators such as the MDD-W elicits a score: 2, 3, 6. Positive changes in these numbers attributable to a certain intervention will tell at the macro level whether things are improving. However, this information will not contribute to improving the quality of implementation of a program or identify specific actions needed to improve diet quality of target groups…”*

*Interview with biofortification expert, USA, March 2021.*

Regardless of these limitations, efforts have been made to adapt the MDD-W to capture the contribution of biofortified foods to nutrient intake. CIP participants indicated that they use the MDD-W to assess the change in dietary diversity among WRA attributable to their interventions with OFSP. CIP modified the MDD-W’s scoring system by adding biofortified crops as a unique and independent food group contributing to diet diversity and extending the maximum MDD-W score to 11. Although this modification has not been validated as a proxy measure for assessing vitamin A adequacy, it enables CIP to assess their interventions’ contribution to dietary diversity in specific geographies.

Another potential method to assess the coverage and consumption of biofortified foods described in the SSIs was the Global Diet Quality Score (GDQS), a novel food-based metric that calculates nutrient adequacy and the risk of non-communicable diseases associated with dietary intake. The GDQS is calculated based on the consumption of 25 food groups (16 healthy, seven unhealthy, and two classified as unhealthy when consumed in excess (i.e., red meat and high-fat dairy)) (40). Total scores ≥23 are associated with a low risk of nutrient inadequacy and non-communicable disease risk, whereas scores ≥15 and <23 indicate moderate risk, and scores <15 indicate high risk (40).

As mentioned by one participant, the GDQS metric and its data collection tool [the GDQS mobile application (41)] could be adapted by adding a specific food group, such as biofortified tubers (e.g., OFSP and yellow cassava), and an extra point to the DGQS metric to account for foods consumed from that food group. Another participant explained that the GDQS data collection method uses a 24hR format; thus, it can capture the consumption of any food, including biofortified foods. The participant explained:

“… *Data are collected with a mobile application that stores dietary intake data in an Excel file. The Excel sheet could be easily modified by adding new rows with the foods of interest, uploading the new file to the app, and it would be ready to capture the consumption of specific foods. However, this modification should be validated. Also, the GDQS data collection method is not designed to capture portion sizes of single foods but to estimate quantities of food intake at the food group level.”*

*Interview with an expert on dietary assessment methods, USA, June 2021.*

The two experts on dietary intake methods also explained that modifications to the GDQS metric and data collection method could be used to assess the contribution of biofortified foods to diet quality and to estimate the coverage of biofortified foods, but not to estimate the contribution of foods to micronutrient intake. Yet, some biofortification experts observed that, given the potential variation in micronutrient content of biofortified foods eventually consumed by households, and their coverage, the use of modified diet quality indicators could lead to over-or underestimation of the contribution of biofortified foods to diet quality.

## Compiling a generic ToC for selecting HLI with their metrics, methods, and tools (Step 3)

**FIGURE 2:**
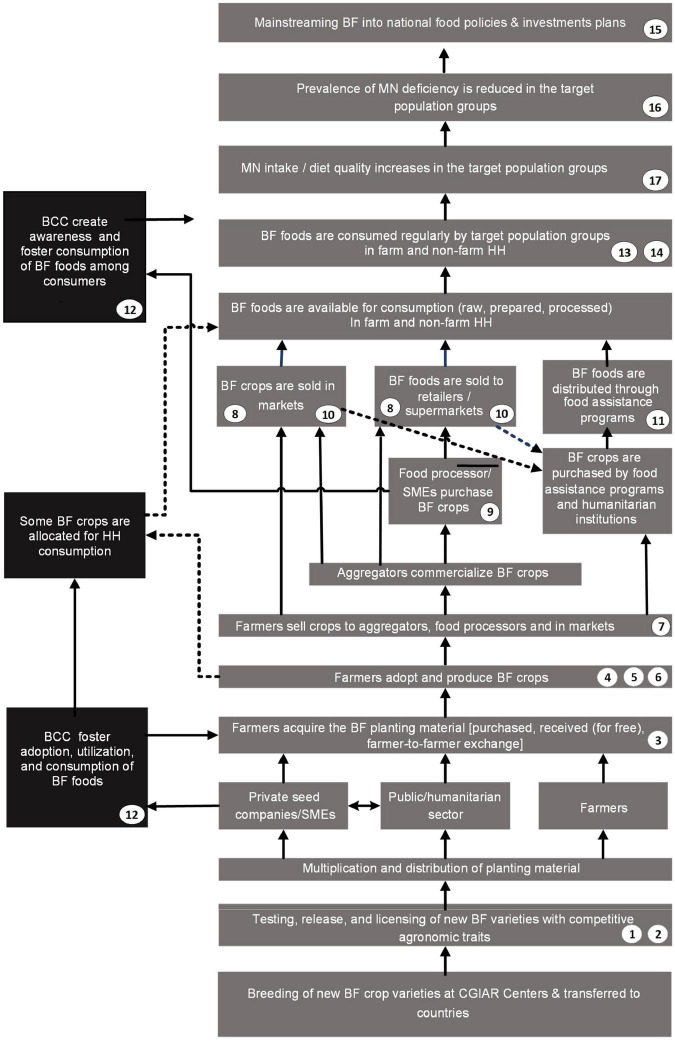
Theory of Change representing the generic biofortification program impact pathway; the numbers in white circles correspond to the indicators shown in Table 6.

Based on the generic description of biofortification programs from the SSI participants (Theme 1) and the internal program documents provided by HarvestPlus and CIP participants (19–25, 33, 42, 43), we compiled the ToC presented in Figure 2. The ToC in Figure 2 deviates from the harmonized MELIAS ToC (23) in two aspects: first, The ToC from Figure 2 visualizes the mainstreaming of biofortification into national policies and investment plans; second, it includes the expected outcome of behavior change communication (BCC) strategies at two levels of the ToC. BCC fosters farmers’ adoption and consumption of biofortified crops at the first level. At the second level, it promotes the consumption of biofortified foods in rural and urban households.

We used the ToC from Figure 2 as an analysis framework to identify 17 HLI for measuring key results along the implementation pathway of biofortification programs. The 17 HLI are presented in Tables 6, 7, along with their definitions and associated metrics, methods, tools, and data sources.

**TABLE 6:** High-level indicators and metrics for M&E of biofortification programs.

No.	Indicator definition	Metric^a^
***Output***		
1	Proportion of crop varieties released that are biofortified^b^ (24)	$\frac{\mathrm{Number}\,\mathrm{of}\,\mathrm{crop}\,\mathrm{varieties}\,\mathrm{released}\,\mathrm{that}\,\mathrm{are}\,\mathrm{biofortified}}{\mathrm{Total}\,\mathrm{number}\,\mathrm{of}\,\mathrm{crop}\,\mathrm{varieties}\,\mathrm{released}}$× 100
2	Proportion of crop varieties with minimum micronutrient level released^c^	$\frac{\mathrm{Number}\,\mathrm{of}\,\mathrm{crop}\,\mathrm{varieties}\,\mathrm{released}\,\mathrm{with}\,\min\,\mathrm{micronutrient}\,\mathrm{level}}{\mathrm{Total}\,\mathrm{number}\,\mathrm{of}\,\mathrm{crop}\,\mathrm{varieties}\,\mathrm{released}}$× 100
***Outcome***		
3	Proportion of farm households^d^ that acquired^e^ biofortified planting material^f^ (24)	Total⁢#⁢of⁢HH⁢(sum⁢of⁢categories⁢ 1⁢to⁢ 3)⁢that⁢acquired⁢biofortified⁢seedTotal⁢number⁢of⁢farmers⁢in⁢that⁢geography× 100
4	Proportion of farm HH^g^ that are growing biofortified crop varieties (24)	$\frac{\mathrm{Total}\,\mathrm{Number}\,\mathrm{of}\,\mathrm{farm}\,\mathrm{HH}\,\mathrm{that}\,\mathrm{planted}\,\mathrm{biofo}\,\mathrm{rtified}\,\mathrm{crops}}{\mathrm{Total}\,\mathrm{number}\,\mathrm{of}\,\mathrm{farm}\,\mathrm{HH}\,\mathrm{in}\,a\,\mathrm{geography}}$× 100
5	Area planted with biofortified crops by farmers^h^ (33)	$\frac{\mathrm{Total}\,\mathrm{area}\,\mathrm{planted}\,\mathrm{with}\,\mathrm{biofortified}\,\mathrm{crop}\,\mathrm{varieties}}{\mathrm{Total}\,\mathrm{area}\,\mathrm{planted}\,\mathrm{by}\,\left[\text{crop}\right]\,\left(\mathrm{biofortified}+\mathrm{non}-\mathrm{biofortified}\right)}$× 100
6	Proportion of crop harvest that is of biofortified varieties (24)	$\frac{\mathrm{Quantity}\,\mathrm{of}\,\mathrm{biofortified}\,\mathrm{crop}\,\mathrm{of}\,\mathrm{interest}\,\mathrm{harvested}}{\mathrm{Total}\,\mathrm{quantity}\,\mathrm{of}\,\mathrm{crop}\,\mathrm{of}\,\mathrm{interest}\,\mathrm{harvested}}$× 100
7	Proportion of farm HH that sell biofortified crops/food^i^ (24)	$\frac{\mathrm{Number}\,\mathrm{of}\,\mathrm{HHs}\,\mathrm{growing}\,\mathrm{and}\,\mathrm{selling}\,\mathrm{biofortified}\,\mathrm{crops}}{\mathrm{Total}\,\mathrm{Number}\,\mathrm{of}\,\mathrm{HHs}\,\mathrm{growing}\,\mathrm{biofortified}\,\mathrm{crops}}$× 100
8	Proportion of raw food in the market that is biofortified^j^ (24)	$\frac{\mathrm{Volume}\,\mathrm{of}\,\mathrm{biofortified}\,\mathrm{food}\,\mathrm{of}\,\mathrm{interest}\,\mathrm{traded}\,\mathrm{in}\,\mathrm{the}\,\mathrm{market}}{\mathrm{Total}\,\mathrm{volume}\,\mathrm{of}\,\mathrm{food}\,\mathrm{of}\,\mathrm{crop}\,\mathrm{of}\,\mathrm{interest}\,\mathrm{in}\,\mathrm{the}\,\mathrm{market}}$× 100
9	Proportion of raw input for processed foods that is biofortified^k^ (24)	$\frac{\mathrm{Total}\,\mathrm{weight}\,\mathrm{of}\,\mathrm{ingredient}\,\mathrm{crop}\,\mathrm{biofortified}}{\mathrm{Total}\,\mathrm{weight}\,\mathrm{of}\,\mathrm{ingredient}\,\left[\text{crop}\right]\,b\,i\,o+n\,o\,n-b\,i\,o\,f\,o\,r\,t\,i\,f\,i\,e\,d}$× 100
10	Proportion of prepared or processed food products available in the market that contain a biofortified food^l^ (24)	$\frac{\begin{array}{c} \mathrm{Number}\,\mathrm{of}\,\mathrm{prepared}\,\mathrm{or}\,\mathrm{processed}\,\mathrm{food}\,\mathrm{products}\,\mathrm{made}\,\mathrm{from}\,\mathrm{the}\,\mathrm{food}\\ \mathrm{vehicle}\,\mathrm{that}\,\mathrm{is}\,\mathrm{confirmed}\,\mathrm{to}\,\mathrm{be}\,\mathrm{biofortified}\,\mathrm{according}\,\mathrm{to}\,\mathrm{the}\,\mathrm{national}\,\mathrm{standard} \end{array}}{\mathrm{Number}\,\mathrm{of}\,\mathrm{all}\,\mathrm{available}\,\mathrm{prepared}\,\mathrm{or}\,\mathrm{processed}\,\mathrm{food}\,\mathrm{products}\,\mathrm{of}\,a\,\mathrm{biofortified}\,\mathrm{food}}$× 100
11	Proportion of staple foods in institutional food distribution programs that is biofortified^m^ (24)	$\frac{\mathrm{Total}\,\mathrm{biofortified}\,\mathrm{foods}\,\mathrm{distributed}}{\mathrm{Total}\,\mathrm{food}\,\mathrm{distributed}\,\left(\mathrm{biofortified}+\mathrm{non}-\mathrm{biofortified}\right)}$× 100
12	Proportion of people that are aware of biofortified crops/foods^n^ (24)	$\frac{\mathrm{Total}\,\mathrm{number}\,\mathrm{people}\,\mathrm{that}\,\mathrm{have}\,\mathrm{ever}\,\mathrm{heard}/\mathrm{seen}\,\mathrm{biofortified}\,\mathrm{crop}/\mathrm{food}}{\mathrm{Total}\,\mathrm{count}\,\mathrm{of}\,\mathrm{people}\,\mathrm{in}\,a\,\mathrm{study}\,\mathrm{area}}$× 100
13	Proportion of individuals in target population group who consumed (in any amount) the biofortified food^o^ (24)	$\frac{\begin{array}{c} \mathrm{No}\,\mathrm{of}\,\mathrm{individuals}\,\mathrm{in}\,\mathrm{target}\,\mathrm{population}\,\mathrm{group}\,\mathrm{that}\,\mathrm{reported}\,\mathrm{consuming}\\ \mathrm{the}\,\mathrm{biofortified}\,\mathrm{food}\,\mathrm{or}\,\mathrm{product}\,\mathrm{that}\,\mathrm{is}\,\mathrm{confirmed}\,\mathrm{to}\,\mathrm{be}\,\mathrm{biofortified}\,\left(\mathrm{in}\,\mathrm{any}\,\mathrm{amount}\right) \end{array}}{\mathrm{Total}\,\mathrm{number}\,\mathrm{of}\,\mathrm{individuals}\,\mathrm{surveyed}}$× 100
14	Amount of the biofortified food consumed daily among target population group^p^ (24)	Reported in average consumption of the biofortified foods in grams. One of the following three consumption assessment methods may be used: apparent consumption based on adult male equivalents, food frequency questionnaires, or 24h recalls
15	Number of policy/plan documents that mention biofortification as a strategy for addressing micronutrient deficiency^q^ (24)	No. of documents per type of document
***Impact***		
16	Proportion of individuals in target population group whose micronutrient intake status shifts from inadequate to adequate due to the consumption of biofortified food(s) over time (24)	$\frac{\begin{array}{c} \mathrm{Total}\,\mathrm{number}\,\mathrm{of}\,\mathrm{people}\,\mathrm{with}\,\mathrm{inadequate}\,\mathrm{micronutrient}\,\mathrm{intake}\,\mathrm{before}\\ \mathrm{and}\,\mathrm{after}\,\mathrm{starting}\,\mathrm{the}\,\mathrm{program} \end{array}}{\mathrm{Total}\,\mathrm{number}\,\mathrm{of}\,\mathrm{people}\,\mathrm{in}\,\mathrm{an}\,\mathrm{intervention}\,\mathrm{area}}$× 100
17	Change in prevalence of Dietary Diversity or Diet Quality Score between baseline and end of project (25)	$\frac{\begin{array}{c} \mathrm{Total}\,\mathrm{number}\,\mathrm{of}\,\mathrm{people}\,\mathrm{with}\,\mathrm{adequer}\,\mathrm{dietary}\,\mathrm{diversity}\,\mathrm{or}\\ \mathrm{diet}\,\mathrm{quality}\,\mathrm{soceres}\,\mathrm{before}\,\mathrm{and}\,\mathrm{after}\,\mathrm{starting}\,\mathrm{the}\,\mathrm{program} \end{array}}{\mathrm{Total}\,\mathrm{number}\,\mathrm{of}\,\mathrm{people}\,\mathrm{in}\,\mathrm{an}\,\mathrm{intervention}\,\mathrm{area}}$× 100

^a^Defined as a set of numbers that give information about a particular process or activity.

^b^This Indicator is disaggregated by crop, geographic location (country), and recipient’s gender, if applicable.

^C^This Indicator focuses on the number of varieties that have been biofortified to a minimum recommended level required to have the expected impact for correcting micronutrient deficiencies. It was recommended by three experts on BF programming from HarvestPlus.

^d^An individual registered as having acquired planting material is assumed to represent a farming household.

^e^“Acquired” refers to planting material distributed, given, or sold to farmers. There are three ways of acquiring seed depending on the source and means of payment: (1) Farmers can pay cash or barter trade to get seed from the seed market; (2) farmers can receive free seed as promotional packs; and (3) Farmers can get recycled seed from fellow farmers voluntary, i.e., farmer to farmer sharing, or obligated sharing, e.g., payback.

^f^This Indicator considers any material (certified, truthfully labeled, quality guaranteed, or recycled) planted as a seed to produce biofortified food crops.

^g^HH, Households.

^h^The information on the area planted may be collected in different ways: (1) self-reported (by the farmer) crop area; (2) area planted calculated using the quantity of seed planted and seed rate (e.g., 160 kg of hybrid rice seed/hectare); (3) field measurements using GPS technology.

^I^This Indicator is constructed with information on the number of households within a geographic area that grows biofortified crops and sell them on their farms, roadside, in markets, to aggregators, or directly to public or private institutions, such as government programs, supermarkets, and food processors, among others.

^j^This Indicator focuses only on raw unprocessed biofortified foods (e.g., grains, cereals, roots, beans). It captures the amount sold or traded (e.g., in kg, tons) of a biofortified crop from programs within specific geographic units.

^k^This Indicator is used to track processed foods (e.g., cooked, canned, frozen, packaged, or foods changed in nutritional composition through food preservation or food preparation) that use biofortified foods as ingredients in substitution of conventional foods.

^l^This Indicator can be used to assess the quality of biofortified foods.

^m^This Indicator monitors institutional commitment to the use of biofortified foods in food assistance, emergency, and safety nets programs as a proportion of the total food distributed through these programs.

^n^This Indicator tracks the proportion of farmers and consumers that have ever heard, seen, or used biofortified crops and foods.

^o^This Indicator tracks the coverage of biofortified foods among targeted population groups.

^p^This Indicator tracks the coverage of biofortified foods among targeted population groups and the nutritional impact attributable to the foods.

^q^Simple count of the number of policies, plans (including investment plans), and programs, that consider biofortification as a strategy.

**TABLE 7:** Summary of methods and tools for constructing the set of high-level indicators.

No.	Indicators	Methods	Tools
1	Proportion of crop varieties released that are biofortified (24)	Simple count of crop varieties that are officially recorded as released (with release certificate) in the current reporting year	● Released varieties checklist
2	Proportion of crop varieties with minimum micronutrient level released	Simple count of crop varieties that are officially recorded as released varieties that meet globally acceptable micronutrient content	● Released varieties checklist
3	Proportion of farm households that acquired biofortified planting material (24)	**Listing survey** The listing survey enumerates the farmers in strategic geographic units that acquired the biofortified planting material through different delivery channels (e.g., seed markets, farmer-to-farmer, public or public/private distribution). The amount and type of biofortified planting material acquired from each delivery channel (e.g., private, public/private, farmer-to-farmer) and the number of farmers growing them are also registered	● Planting material distribution form ● Planting material payback/pass-on form ● Multiplier registration form ● Demonstration plot establishment form ● Electronic M&E database
4	Proportion of farm HH that is growing biofortified crop varieties (24)		
5	Area planted with biofortified crops by farmers (33)	**Main survey** The main survey assesses farmers’ behavior toward biofortified crops, e.g., crop production and commercialization, as well as awareness and consumption of biofortified foods. The main survey is undertaken at the end of a cropping season, and it includes data collection on household composition	● Paper-based or electronic survey forms ● Electronic M&E database
6	Proportion of crop harvest that is of biofortified varieties (24)		
7	Proportion of farm households that sell biofortified crops/food (24)		
12	Proportion of people that are aware of biofortified crops/foods (24)		
8	Proportion of raw food in the market that is biofortified (24)	**Market survey** It can be undertaken in fresh produce markets and/or supermarkets. Review of food processors’ records to track substitution of low nutrient-dense with high nutrient dense biofortified ingredients (measured in g, kg, or MT)	● Paper-based or electronic survey forms ● Electronic M&E database
9	Proportion of raw input for processed foods that is biofortified (24)		● Food processor’s records ● Electronic M&E database
10	Proportion of prepared or processed food products available in the market that contain a biofortified food (24)	**Market assessment** Count all prepared or processed food products available in the market made from biofortified and non-biofortified varieties. For crops with visible trains, a visual assessment can be made. For crops with invisible traits, biofortification needs to be verified in a laboratory	● Paper-based or electronic survey forms ● Electronic M&E database
11	Proportion of staple foods in institutional food distribution programs that is biofortified (24)	Review of institutional data from government or other implementing partners. Those data may be in the form of national statistics on institutional seed distribution or registered in records of implementing Institutions	● Electronic M&E database
13	Proportion of individuals in target population group who consumed (in any amount) the biofortified food (24)	**Coverage and consumption assessment methods** ● Individual dietary intake assessment: 1.24-hour recall 2. Food Frequency Questionnaire ● Modeling: Coverage estimates from monitoring/adoption surveys are used to estimate the consumption of biofortified food. This method uses the coverage rate of a particular biofortified crop (from listing surveys), dietary intake data, and nutrient composition data of the crop. Individual dietary intake of specific food can be estimated through the Adult Male Equivalent method, starting from household–level consumption data. Household-level consumption can be collected through, e.g., monitoring surveys (main survey) or existing national-level Household Consumption and Expenditure Surveys (HCES)^a^.	● Paper-based or electronic survey forms ● Electronic M&E database
14	Amount of the biofortified food consumed daily among target population group (24)		
16	Proportion of individuals in target population group whose micronutrient intake status shifts from inadequate to adequate due to the consumption of biofortified food(s) over time (24)		
17	Change in prevalence of Dietary Diversity or Diet Quality Score between baseline and end of project (25)	Dietary diversity and/or quality scores collected through pre-tested and/or validated surveys, e.g., Minimum Dietary Diversity Score for Women (MDD-W)	● Paper-based or electronic Diet Diversity/Quality Score survey forms ● Electronic M&E database
15	Number of policy/plan documents that mention biofortification as a strategy for addressing micronutrient deficiency (24)	Snapshot survey applied to relevant authorities and received means of verification documents to perform a simple count of the number of policies, plans (including investment plans), and programs that consider biofortification as a strategy.	● Electronic M&E database

^*a*^Household Consumption and Expenditure Surveys (HCESs) are a group of socioeconomic surveys such as Household Income Expenditure Surveys (HIES), Living Standards Measurement Studies (LSMS), National Household Budget Surveys (NHBS), among others, that collect detailed food consumption data. Those surveys are available in most LMIC, and data are collected regularly. When HCES data are combined with food composition data, they can be used to estimate the micronutrient supply at the household level (54).

## Actionable recommendations

**FIGURE 3:**
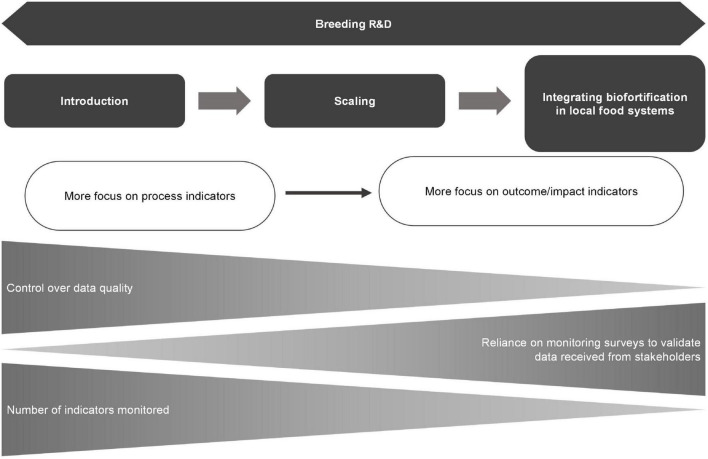
Evolution of the M&E framework across biofortification implementation phases.

A generic or global M&E framework for biofortification programs with a comprehensive set of 17 high-level indicators is presented with their respective metrics, methods, and tools for data collection. The framework is developed based on the commonalities of biofortification programs implemented in multiple LMICs in Africa, Asia, Latin America, and the Caribbean regions by collaborations between national and international agricultural research systems and other public, private, NGO, and UN stakeholders. The 17 HLI presented in this paper may not be used all at once. Their use is flexible and can be prioritized, depending on the implementation stage and stakeholders’ information needs, as illustrated in Figure 3. Like any other program, biofortification programs mature through their different implementation stages, e.g., from the breeding phase to the introduction of biofortified crops and the scaling phase. Along this pathway, the indicators in use may shift from an initial focus on program outputs to a later focus on outcomes and impact. The required number of indicators may also change, as the scaling phase will require cost-efficient prioritization of M&E activities.

This study highlights crop-related differences in biofortification programming. Competitive planting materials such as grain crop seeds for hybrid and OPV are usually licensed to commercial private or public/private seed companies. They are distributed to farmers through formal commercial channels. VPCs such as OFSP or cassava belong to decentralized informal seed systems, where the planting material is exchanged/shared with other farmers or sold in local markets by traders and vendors (44). Yet, the crop-related differences in biofortification programming should not be an obstacle to using a generic or global M&E framework, particularly at the outcome level. However, M&E of biofortification programs with informal seed systems will require active involvement and investment of the private sector and NGOs, while monitoring programs with formal seed systems will require joint efforts of private seed companies, NGOs, and governments (45).

Sentinel site surveys may be a convenient alternative for M&E of biofortification interventions of formal and informal seed systems. They can generate timely actionable information to improve program implementation at a relatively low cost (46, 47). Sentinel site surveys have long been used in multiple nutrition and health interventions (46–48). The government of Costa Rica, for example, uses them to track changes in iron deficiency and anemia attributable to their food fortification programs (47). It is undertaken in strategically selected urban and rural areas (47). HarvestPlus SSI participants also indicated they use sentinel site surveys as an option to monitor program outcomes in targeted geographies. Nevertheless, for mature programs with high coverage, most participants recommended the integration of biofortified crops and foods into production and consumption modules of existing nationally representative data collection systems, e.g., National Crop Surveys, Nutrition, and Health Surveys, HCES, or Demographic and Health Surveys. This would ensure cost-effective, regular, and sustainable data collection on the production, coverage, and consumption of biofortified crops and foods and contribute to monitoring diet quality (12).

Concerning the 17 HLI, Most of them have already been tested globally in multiple biofortification projects (indicators 1, 3-7, 12, 13, and 17). Additionally, indicators 1, 3, 4, and 6–16 were anchored to the ToC developed by the MELIAS collective, and hence their definitions are now harmonized across biofortification programs led by HarvestPlus, CIP, and GAIN. Furthermore, the indicators on awareness (indicator 12), coverage, and consumption (indicator 13) were built on approaches previously used to assess large-scale food fortification programs (LSFF) (34, 49). Indicator 2 for monitoring the proportion of biofortified varieties released with minimum micronutrient content was recommended by three SSI participants to establish a parameter for monitoring the quality of biofortified planting material. Some countries have already included minimum micronutrient levels as criteria for releasing biofortified varieties; e.g., the government of India established a minimum standard for levels of iron (42 ppm) and zinc (32 ppm) for all released pearl millet varieties (10). However, standards for seed biofortification levels still need to be defined/implemented in most countries. Although this indicator has not been tested, we believe that it will be crucial to enforce compliance of private and public seed companies with biofortification levels in the near future. Monitoring compliance is also crucial in ensuring the quality of fortified food in LSFF programs (6, 50).

Likewise, indicators 8–11 for assessing market availability of raw, prepared, and processed biofortified foods were recently developed by HarvestPlus and GAIN for the scaling phase of biofortification programs and are not tested. Proposals for studies to test these indicators are being prepared. Indicator 17 (i.e., change in the prevalence of dietary diversity or diet quality scores between baseline and endline) can be constructed using a diet quality indicator, e.g., the MDD-W or GDQS. These indicators are most useful once significant coverage of biofortified crops and foods is attained and when consistent data on the quality of biofortified crops and foods (i.e., level of biofortification meeting target ranges) are available. Using this indicator with uncertain data on coverage and biofortification quality may lead to under-or overestimating the contribution of biofortified foods to diet quality.

The generic ToC presented in Figure 2 permits visualizing crucial stages of biofortification programs that can be monitored with the 17 HLI. However, it should be noted that other indicators may be needed to track specific inputs and activities at project-level management along the implementation cycle. We aimed to identify HLI to enable common data collection across geographies, institutions, and programs to enable aggregation and comparison of results. Further research is recommended to test, revise, and harmonize this framework with indicators across programs, implementing institutions, and countries as biofortification programs are scaled across and within LMIC.

## Discussion

This study presents a generic M&E framework for biofortification programs, including a ToC for a generic or ‘global’ biofortification program impact pathway and a set of 17 HLI and their associated methods, metrics, and tools. The study is based on a thorough review of a wide range of available frameworks from published and gray literature and ground-truthed through interviews with experts in biofortification programming and dietary intake assessment.

The generic implementation pathway of biofortification programs described in Theme 1 and represented in Figure 2 is a compilation of descriptions of current biofortification programs described by the participants interviewed for this study and a harmonized version of the following: generic ToC for biofortified crops (19), a generic ToC for cassava (20), two country-specific ToCs for cassava and maize for Nigeria (21) and Zambia (22), and the ToC developed by the MELIAS group (23). The latter represents a generic description of the implementation pathways of biofortification programs at scale, harmonized across HarvestPlus, CIP, and GAIN-led biofortification programs, and it is grounded in cross-country, multi-year program experiences of these institutions and their partners in Africa, Asia, and Latin America and the Caribbean.

Compared to the ToC of the MELIAS collective (23), the ToC from Figure 2 differs in two aspects. First, it visualizes the integration of biofortification into national policies and investment plans, which can be instrumental for the sustainable scaling of biofortification programs. Second, it visualizes the expected changes in behavior toward adopting biofortified crops and foods among farmers and consumers—of urban and rural settings—attributable to BCC. From our perspective, BCC strategies will be a crucial element of biofortification programming to foster the adoption and consumption of biofortified crops and foods at all levels of the value chain.

This study has limitations and strengths. A limitation is that most of the information on the M&E framework and biofortification programming was obtained from program documents provided by HarvestPlus and CIP. Though these two organizations have been spearheading biofortification programming in the past decade, relying on only their points of view may represent a narrow scope. Although we searched for biofortification programs implemented by other organizations, we couldn’t find many, and those we found did not provide significant relevant information for the aims of this study. The joining of GAIN of HarvestPlus’s efforts in 2018 to scale biofortified foods (51, 52) and the most recent national uptake by governments, NARS, NGOs, and associated partners will likely broaden the number and types of institutions implementing biofortification programs in the coming years. Once that happens, the M&E framework for biofortification programs presented warrants a revisit, and until then, the framework presented here can be used by biofortification programmers.

The main strengths of this study include (1) compilation of a generic ToC for biofortification programming based on a review and triangulation of tried-and-tested ToCs of various biofortification programs implemented globally; (2) identification of the most pertinent HLI used by biofortification programs and anchoring of these to the ToC above; and (3) review of available IMMT to recommend a sub-set of HLI and ground-truthing of these with experts on biofortification programming. This multi-method iterative approach allowed us to identify rich information on biofortification programs to address our research aims.

Further work is required to determine how IMMT for biofortification programs can best be incorporated into existing national data collection systems and integrated into other nutrition and health indicator-based high-level reporting such as Demographic and Health Surveys. Furthermore, implementation research (e.g., formative research, process evaluation) is warranted to explore barriers and enablers to using the proposed HLI in ongoing biofortification programs, including programs implemented in remote areas with difficult access. This will help to further harmonize this framework across programs, countries, geographies, crops, and institutions; and to optimize planning and funding of monitoring and evaluation activities to ensure that biofortification programs reach the neediest.

Another area of research that we suggest implementing is in the artificial intelligence field. Participants unanimously described numerous limitations to implementing food consumption surveys using traditional methods such as 24hR and FFQ. Artificial intelligence applications could help simplify these assessments, for example, by using pictures captured with a mobile phone integrated into an artificial intelligence-based food recognition system and linking them to mega-databases of food composition tables (55) to estimate food intake and nutrient composition, respectively. This area of research is in line with the United Nations’ 17 Sustainable Development Goal and the Artificial Intelligence for Social Good movement (56). Yet any artificial intelligence initiative should follow ethical principles and guidelines for developing innovative and trustworthy technologies.

## Author contributions

SR-M participated in research design, data collection and analysis, and conceptualizing the manuscript. FG participated in data collection and analysis and wrote the manuscript with SR-M, BM, and EB shared the gray literature, ToCs, and indicators used by HarvestPlus globally, participated in SSIs, and critically reviewed the manuscript. SK and CL advised on the study design and critically revised the manuscript. TA developed the syntax for the mapping review, participated in the mapping review data analysis, and critically revised the manuscript. AM-B was the current project manager and contributed to the manuscript. KH was the former project manager and contributed to implementing the project. IB and SO developed the proposal to obtain funding for this study. EF critically reviewed the research proposal and manuscript. SR-M and FG had primary responsibility for the final content. All authors read and approved the final version of the manuscript.

## Abbreviations

24hR: 24-hour Recalls
2FAS: Food Fortification Advisory Services funded by the European Union
CIP: International Potato Center
FAO: Food and Agriculture Organization of the United Nations
FFQ: Food Frequency Questionnaire
GAIN: Global Alliance for Improved Nutrition
GDQS: Global Diet Quality Score
HCES: Household Consumption and Expenditure Surveys
HH: Household
HLI: High-level indicators
IMMT: Indicators, Methods, Metrics and Tools
LMIC: Low- and Middle-Income Countries
LSFF: Large-Scale Food Fortification
M&E: Monitoring & Evaluation
MDD-W: Minimum Dietary Diversity Score for Women
NARS: National Agricultural Research System
NGO: Non-Governmental Organization
OFSP: Orange Fleshed Sweet Potato
OPV: Open Pollinated Variety
SPV: Self-pollinating Variety
VPC: Vegetatively Propagated Crop
SSI: Semi-Structured Interview
ToC: Theory of Change
WRA: Women of Reproductive Age.
